# Transcriptomic and physiological analysis of common duckweed *Lemna minor* responses to NH_4_^+^ toxicity

**DOI:** 10.1186/s12870-016-0774-8

**Published:** 2016-04-18

**Authors:** Wenguo Wang, Rui Li, Qili Zhu, Xiaoyu Tang, Qi Zhao

**Affiliations:** Biogas Institute of Ministry of Agriculture, Section 4-13, Renmin Road South, Chengdu, 610041 Sichuan PR China; Faculty of Biotechnology Industry, Chengdu University, 1 Shiling Street, Chengluo Road, 610106 Chengdu, Sichuan PR China; Key Laboratory of Development and Application of Rural Renewable Energy, Ministry of Agriculture, Section 4-13, Renmin Road South, Chengdu, 610041 Sichuan PR China

**Keywords:** *Lemna minor*, NH_4_^+^ toxicity, RNA-seq, Transcriptome, Oxidative damage, Lignin biosynthesis, Phenylpropanoid pathway, Oxidative damage, Programmed cell death

## Abstract

**Background:**

Plants can suffer ammonium (NH_4_^+^) toxicity, particularly when NH_4_^+^ is supplied as the sole nitrogen source. However, our knowledge about the underlying mechanisms of NH_4_^+^ toxicity is still largely unknown. *Lemna minor*, a model duckweed species, can grow well in high NH_4_^+^ environment but to some extent can also suffer toxic effects. The transcriptomic and physiological analysis of *L. minor* responding to high NH_4_^+^ may provide us some interesting and useful information not only in toxic processes, but also in tolerance mechanisms.

**Results:**

The *L. minor* cultured in the Hoagland solution were used as the control (NC), and in two NH_4_^+^ concentrations (NH_4_^+^ was the sole nitrogen source), 84 mg/L (A84) and 840 mg/L (A840) were used as stress treatments. The NH_4_^+^ toxicity could inhibit the growth of *L. minor*. Reactive oxygen species (ROS) and cell death were studied using stained fronds under toxic levels of NH_4_^+^. The malondialdehyde content and the activities of superoxide dismutase and peroxidase increased from NC to A840, rather than catalase and ascorbate peroxidase. A total of 6.62G nucleotides were generated from the three distinct libraries. A total of 14,207 differentially expressed genes (DEGs) among 70,728 unigenes were obtained. All the DEGs could be clustered into 7 profiles. Most DEGs were down-regulated under NH_4_^+^ toxicity. The genes required for lignin biosynthesis in phenylpropanoid biosynthesis pathway were up-regulated. ROS oxidative-related genes and programmed cell death (PCD)-related genes were also analyzed and indicated oxidative damage and PCD occurring under NH_4_^+^ toxicity.

**Conclusions:**

The first large transcriptome study in *L. minor* responses to NH_4_^+^ toxicity was reported in this work. NH_4_^+^ toxicity could induce ROS accumulation that causes oxidative damage and thus induce cell death in *L. minor*. The antioxidant enzyme system was activated under NH_4_^+^ toxicity for ROS scavenging. The phenylpropanoid pathway was stimulated under NH_4_^+^ toxicity. The increased lignin biosynthesis might play an important role in NH_4_^+^ toxicity resistance.

**Electronic supplementary material:**

The online version of this article (doi:10.1186/s12870-016-0774-8) contains supplementary material, which is available to authorized users.

## Background

Ammonium (NH_4_^+^) and nitrate (NO_3_^−^) are the two inorganic nitrogen (N) forms that can be directly absorbed by plants [1]. Compared to NO_3_^−^, NH_4_^+^ is more easily absorbed by plants as its assimilation requires less energy. But in fact, only few plants are known to be NH_4_^+^ specialists, most of high plants are usually sensitive to NH_4_^+^ [2, 3]. Non-specialists could display toxicity symptoms such as leaf chlorosis, growth suppression, yield depressions, and even mortality in high NH_4_^+^ conditions, particularly when NH_4_^+^ is supplied as the sole N source [3]. At the ecosystem level, some studies have even shown that increased NH_4_^+^ in soil and water environment was associated with reduced crop yield, and decline of forest and macrophyte abundances [4–6].

NH_4_^+^ toxicity is not only a significant ecological issue, but also an important plant physiological process [7]. Plant scientists have been trying to reveal its occurrence, signal transmission and physiological targets in plants [3, 7, 8]. Usually, for most plants, the root bears the brunt of NH_4_^+^ toxicity [7, 9–11]. The root often accumulates high levels of NH_4_^+^ in high NH_4_^+^ condition, and the root cells could experience a futile transmembrane NH_4_^+^ cycling that could carry high energetic cost resulting to decline plant growth [9, 12–14]. Associated enzymes involved in the regulation of NH_4_^+^ influx, a signaling pathway model under NH_4_^+^ toxicity in Arabidopsis thaliana has been described [7, 8]. Additionally, recent studies also revealed that the NH_4_^+^ toxicity could break the intracellular pH balance and C/N balance [3, 7, 15], and cause oxidative damage [16, 17].

The knowledge on NH_4_^+^ toxicity has greatly expanded in recent years, but the underlying mechanism are still largely unclear, further researches, especially in the subcellular level, using more advanced -*omics* approaches to follow up NH_4_^+^-induced global changes in plants are also required [8, 18]. Transcriptome analysis is an effective method for global expression profiling of genes involved in stresses and toxicity in living organisms [19, 20]. For example, transcriptomic profiling using microarrays have been used in Arabidopsis to identify molecular changes involved in NH_4_^+^ toxicity [21]. With the rapid development of high-throughput sequencing, the next-generation transcriptome profiling approach or RNA sequencing (RNA-seq) has been gaining wide attention and use. RNA-seq could provide more information at a more affordable cost compared with the microarray and now an emerging powerful tool for transcriptome analysis [22].

Duckweeds are simple floating aquatic plants composed by frond and root. It has been considered to be a model species for aquatic plants and has been greatly used previously especially in the fields of toxicity studies, phytoremediation and biofuels production [23]. *Lemna minor* L. is one of the most widely distributed duckweed species and gains increasing interests due to its better adaptability to varying environmental conditions including high NH_4_^+^ stress [24, 25]. *L. minor* could grow well in high NH_4_^+^ environment and has been even recognized as ‘NH_4_^+^ specialist’, but has been shown to still suffer toxicity in very high NH_4_^+^ levels [15]. On the other hand, mechanisms and processes of toxicity in duckweeds however are a bit different from the terrestrial plant. Such as in Arabidopsis, most of the NH_4_^+^ contact happens mainly in roots, thus the roots firstly suffer NH_4_^+^ toxicity [7, 26]. While for the floating duckweeds, the frond and root are all directly exposed to the toxic environment. This may lead to some different responses from the terrestrial plant. In this study, we use RNA-seq to investigate the global changes in common duckweed *Lemna minor* under NH_4_^+^ toxicity. Those transcriptome analyses may provide some interesting insights and useful information not only in intoxication processes, but also on its potential tolerance mechanisms.

## Methods

### Sample preparation

Samples were prepared as described in Wang et al. [15]. *L. minor* was collected from a eutrophic pond in Chengdu, Sichuan, China (location: 30° 38.86′N, 104° 18.01′ E; elevation 499 m), and no specific permissions were required for specimen collection. To guarantee genetic uniformity, all of the *L. minor* materials originated from single colony and cultivated in Hoagland solution with 84 mg/L NO_3_^−^. The *L. minor* cultured in the Hoagland solution were used as the control (NC). For the treatments, cultures were grown in two NH_4_^+^ concentrations, 84 mg/L (A84) and 840 mg/L (A840) in improved Hoagland solution, in which NH_4_Cl was used to provide NH_4_^+^, and KCl and CaCl_2_ were used to replace KNO_3_ and Ca(NO_3_)_2_ to avoid the impact of nitrate. All the solutions used in this study were adjusted to pH 5.5 with 1 M HCl.

Before inoculation, the fronds collected from Hoagland were washed five times with deionized water. Then, 0.2 g (fresh weight) of plant materials was cultivated in plastic basins with water depth of 2 cm. The plants were grown for one week in incubator at 23 ± 1 °C with a photon flux density of 50–60 μmol · m^−2^ · s^−1^ provided by cool white fluorescent bulbs in a 16 h light/8 h dark cycle. The medium in each container was replaced every day.

### Growth and physiological analysis

The relative growth rate (RGR) based on fronds number was used to evaluate the duckweed growth in different treatments as previously described in Wang et al. [15]. A total of 0.5 g fronds homogenized in 5 ml 0.1 % trichloroacetic acid was used for malondialdehyde (MDA) estimation by the thiobarbituric reaction following Dhindsa and Matowe [27]. Superoxide dismutase (SOD) was measured using a kit from Nanjing Jiancheng Bioengineering Institute (Jiangsu, China). Peroxidase (POD) and catalase (CAT) were measured by absorption photometry using a spectrophotometer as described by Bestwick et al. and Aebi [28, 29], respectively. Ascorbate peroxidase (APX) activity assays were according to the method of Chen and Asada where the extinction coefficient of ascorbate at 290 nm was used for calculating APX enzyme activity [30].

Fronds of *L. minor* from the three treatments were stained by 3,3′-diaminobenzidine (DAB) or nitroblue tetrazolium (NBT) for measuring H_2_O_2_ or O_2_^−^ level, respectively [31]. Cell death was examined by Evans blue staining as described by Kim et al. [32].

### RNA extraction, cDNA library preparation and sequencing

The whole plants with fronds and roots were ground in liquid nitrogen and total RNA was extracted using RNeasy® Plant Mini Kit (Qiagen) as per manufacturer’s protocol. The integrity of RNA was assessed by formaldehyde agarose gel electrophoresis. A total of 30 μg mixed RNA from three biological replicates detected by 2100 Bioanalyzer (Agilent, USA) was digested with DNase I (TAKARA), and then purified by Dynabeads® Oligo (dT)25 (Life, USA). 100 ng derived mRNAs were fragmented and reverse transcribed into first-strand cDNAs with random hexamer and then the second-strand cDNAs were synthesized by using a NEBNext® Ultra™ RNA Library Prep Kit for Illumina (NEB). The double-stranded cDNAs were purified and ligated to adaptors for Illumina paired-end sequencing. The cDNA library was sequenced using the Illumina HiSeq2500 system by Shanghai Hanyu Biotech lab (Shanghai, China).

### *De novo* assembly of RNA-seq reads and quantifying gene expression

For the assembly library, raw reads were filtered using the FASTX-Toolkit (http://hannonlab.cshl.edu/fastx_toolkit/) to remove adapters and low-quality reads (base quality < 20, read length < 40 bp). The obtained quality-filtered reads were *de novo* assembled into contigs by the Trinity Program [33]. Unigenes were defined after removing redundancy and short contigs from the assembly. The unigenes were predicted by “GetORF” in the EMBOSS package [34] and aligned to the protein sequence database NCBI NR (non-redundant protein database), Swiss-Prot (Annotated protein sequence database), KEGG (Kyoto encyclopedia of genes and genomes) and COG (Clusters of orthologous groups of protein) by Blastp with an E-value threshold of 1 × 10 ^−5^.

The number of unique-match reads was calculated and normalized to RPKM (reads per kb per million reads) for gene expression analysis. Comparison of unigene expression between treatments was according to DESeq as described by Abders and Huber [35]. The differentially expressed genes (DEGs) between NC and A84, or between NC and A840, or between A84 and A840 were restricted with FDR (false discovery rate) ≤ 0.001 and the absolute value of log2 Ratio ≥1.

To examine the expression profile of DEGs, the expression data υ (from NC, A84 and A840 treatment) were normalized to 0, log2 (υ_A84_/υ_NC_), log2 (υ_A840_/υ_NC_), and then clustered by Short Time-series Expression Miner software (STEM) [36]. The clustered profiles with *p*-value ≤ 0.05 were considered as significantly expressed. Then the DEGs in all or in each profile were subjected to gene ontology (GO) classifications using WEGO [37], and KEGG pathway enrichment analysis.

### Validation of differential expression using qRT-PCR

The cDNA was generated from 1 μg total RNA isolated from the fronds using a Prime-Script™ 1st Strand cDNA Synthesis Kit (TAKARA, Japan). Primers for quantitative real time PCR (qRT-PCR) were designed using Primer Premier 5.0 software (Premier, Canada) and synthesized by Sangon Biotech (Shanghai) Co., Ltd. The 18S (GenBank accession number: KJ400889) was selected as reference. All the primers are shown in Additional file 1: Table S1. qRT-PCR was performed on a Bio-Rad iQ5 Optical System Real Time PCR System (Bio-Rad, USA). Each reaction mixture was 20 μL containing 10 μL of SYBR Green PCR Master Mix (TaKaRa, Japan), 250 nM of each primer, and 6 μL of diluted first-strand cDNAs. The qRT-PCRs were run as follows: 50 °C for 2 min, 95 °C for 10 min, followed by 40 cycles of 95 °C for 30 s, 56 °C for 30 s, and 72 °C for 30 s in 96-well optical reaction plates. The Ct values were determined for three biological replicates, with three technical replicates for each value. Expression levels of the tested reference genes were determined by Ct values and calculated by 2^-△△Ct^.

### Statistical analysis

All data were statistically analyzed by means of the SPSS with LSD to identify differences. Significant differences (*P* < 0.05) between treatments are indicated by different letters.

## Results

### Phenotypic and physiological responses to NH_4_^+^ toxicity in *L. minor*

**Fig. 1:**
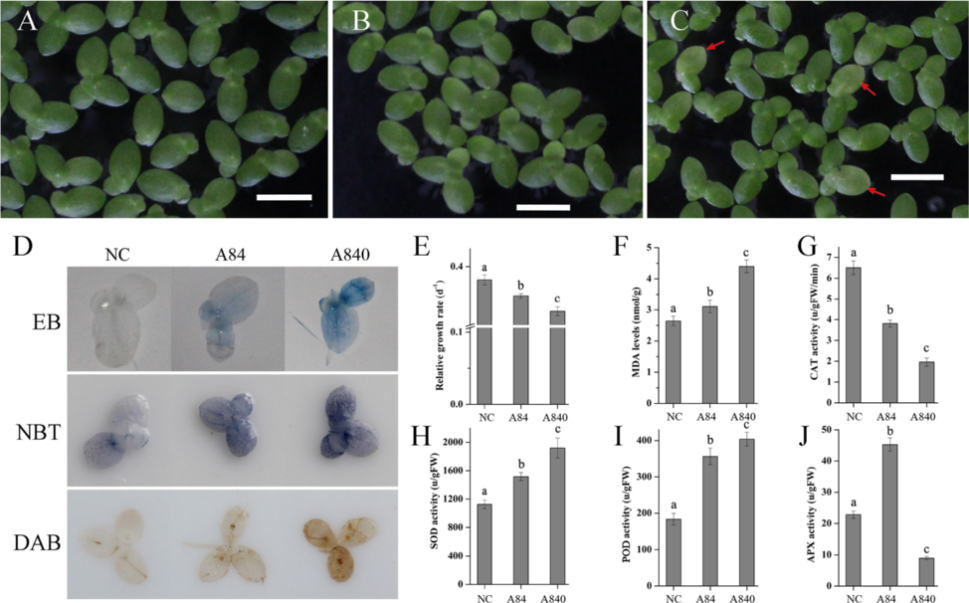
Phenotypic and physiological responses of *Lemna minor* in NC, A84 and A840. **a**-**c**, the appearance of *L. minor* in NC, A84 and A840, respectively, red arrows showed the greensick fronds, scale bar 5 mm; **d** Histochemically staining of cell death, O_2_^−^ and H_2_O_2_ by Evans blue, nitroblue tetrazolium (NBT) and 3,3′-diaminobenzidine (DAB), respectively; **e** relative growth rate (RGR) based on fronds number; **f** MDA contents; **g**-**j**, enzyme activity of catalase (CAT), superoxide dismutase (SOD), peroxidase (POD) and ascorbate peroxidase (APX), respectively

Figure 1 a-c shows changes in the appearance of *L. minor* fronds at the end of experiment. The fronds in NC looked green and healthy, as well as in A84. But in A840, some mother fronds looked greensick (Fig. 1 c, shown by arrow). The RGR based on fronds number showed a downward trend from NC to A840 (Fig. 1 e). This could indicate that the NH_4_^+^ concentrations of 84 mg/L affected the propagation of *L. minor*, and the much higher concentration of 840 mg/L significantly inhibited the growth and could cause some damage.

Evans blue was used to determine the high NH_4_^+^-stress induced cell death (Fig. 1d). Almost no dead cell was stained in the fronds cultured in NC. Dead cells were however detected in both mother and newborn fronds in the plants grown in both tested NH_4_^+^ concentrations, especially in A840.

Fronds of *L. minor* were stained with DAB or NBT to reveal in situ accumulation of two main reactive oxygen species (ROS), H_2_O_2_ and O_2_^−^, respectively (Fig. 1d). Histochemically stained cells showed that the H_2_O_2_ and O_2_^−^ significantly accumulated in both the mother and newborn fronds in A840 after seven days. The fronds in A84 were also found to have some ROS accumulation. For the fronds in NC, the ROS was just slightly accumulated in some mother fronds that might be induced by the normal ageing.

The MDA content was used to detect the lipid peroxidation and membrane damage induced by oxidative stress. The contents of MDA in *L. minor* in A84 and A840 were higher than in NC, and the highest MDA content reached 4.4 nmol/g in A840 (Fig. 1f). The activity of the antioxidant defense system was also analyzed (Fig. 1g-j). Like the change of MDA, the activities of SOD and POD all increased from NC to A840, and the values all increased almost doubled. In contrast, the CAT decreased from NC (6.5 u/g) to A840 (3.8 u/g). For the APX, the highest activity was in A84 (45.28 u/g) and the lowest one was in A840 (8.96 u/g).

### Overview of three libraries data sets by RNA-seq

As shown in Table 1, a total of 6.62G nucleotides, equivalent to 33,136,337 raw reads and 32,403,455 quality filtered (clean) reads were generated from the three separate libraries from NC, A84, and A840. The RNA-seq generated clean reads ranged from 10.4 to 11.1 million on each sample. The Q20 percentages of the three libraries were from 97.21 to 97.44 %, and the GC contents ranged from 50.68 to 51.69 %. All clean reads were pooled together and then *de novo* assembled by Trinity. Based on chosen criteria, an average of 79.91 % of the clean reads was mapped, with perfect matches were from 47.03 to 47.09 %. In each library, the scales of clean reads uniquely mapped to the database were 76.87, 80.09 and 79.91 %, respectively. There were still approximately 20.09 % of clean reads that cannot be mapped back to any references, which could be due to the limited reference gene database of *L. minor*.

**Table 1:** Throughput and quality of RNA-seq of the three libraries

Libaries	Raw reads	Clean reads	Total nucleotides	Q20 (%)	GC (%)	Total mapped reads (%)	Unique match (%)	Perfect match (%)
NC	11,550,000	11,167,248	2.31G	97.44	50.68	77.84	76.87	47.03
A84	10,617,033	10,409,566	2.12G	97.21	51.55	81.04	80.09	47.71
A840	10,969,304	10,826,641	2.19G	97.22	51.69	80.85	79.91	47.79

The final assembly of *L. minor* had 71,094 contigs with length ≥ 200 bp and after further removal of redundant sequences, 70,728 unigenes were obtained. The length of unigenes ranged from 201 b to 14,857 b, with a mean size of 620 bp and N50 number of 988 bp (Table 2). In NC library, the number of genes identified increased with the number of reads until above 6 million, but 4 million in the other two libraries (Additional file 2: Figure S1A). The unigene coverage analyzed as a means of evaluating the quality of the RNA-seq data was mostly >50 %. More than half of the sequences have coverage more than 80 % (Additional file 2: Figure S1B).

**Table 2:** Summary of assemblies of RNA-seq data

Summary	Contig	Unigene
Total number	71,094	70,728
Average length (bp)	618	620
Min length (bp)	201	201
Max length (bp)	14,857	14,857
N50 length (bp)	988	988

The amino acid sequences predicted by ‘Getorf’ were searched by BLASTP. A final number of 29,171, 28,476, 17,209 and 14,172 unigenes (E-value < 1e^−5^) had significant matches in NR, KEGG and COG databases, respectively. As shown in Additional file 3: Figure S2, the sequences matched with the species in NR were determined as following: 27 % *Vitis vinifera*, 16 % *Ricinus communis*, 14 % *Populus trichocarpa*, 8 % *Glycine max* and 4 % *Oryza sativa* etc. The COG matched *L. minor* unigenes dataset were categorized into 25 functional COG clusters (Additional file 4: Figure S3). The five largest functional categories in which sequences were identified to include 1) Posttranslational modification, protein turnover, chaperones; 2) Signal transduction mechanisms; 3) General function prediction only; 4) Translation, ribosomal structure and biogenesis; and 5) Intracellular trafficking, secretion, and vesicular transport.

### Identification and overview of the differentially expressed genes

**Fig. 2:**
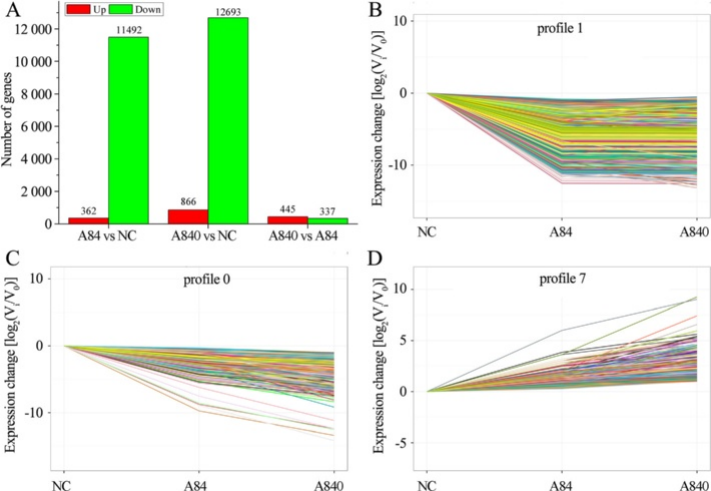
Unigene expression pairwise comparison (**a**) and three main DEGs expression profiles (*p*-value ≤ 0.05) (**b**-**d**) in three libraries (NC, A84 and A840)

We performed a pairwise comparison using NC as the control, and A84, or A840 as the treatments (Fig. 2a). Most of genes were down-regulated in the two treatments but the up-regulated genes in A840 were slightly higher than the down-regulated genes.

Results from FDR identification showed that 14,207 unigenes were classified as DEGs, which were then used for the subsequent analysis. All the 14,207 DEGs could be clustered into 7 profiles by STEM (Additional file 5: Figure S4; Additional file 6), in which 12,959 DEGs were further clustered into 3 profiles (p-value ≤ 0.05), including two down-regulated patterns (Profile 1 and Profile 0) and one up-regulated pattern (Profile 7) (Fig. 2b-d). Profile 1 and Profile 0 contained 11,625 and 954 DEGs, respectively, while Profile 7 contained 380 DEGs.

**Fig. 3:**
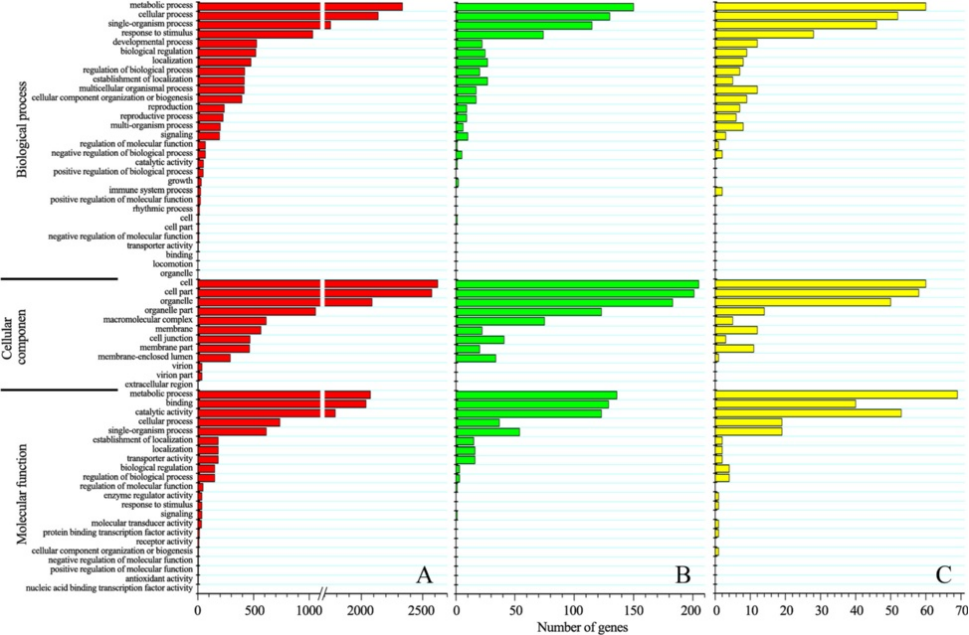
GO classification of profile 1 (**a**), profile 0 (**b**) and profile 7 (**c**) in three libraries (NC, A84 and A840)

Next, the DEGs within the three profiles were subjected to GO-term analysis (Fig. 3). The DEGs were classified into three main categories including cellular component, biological process, and molecular function. Cell and cell parts under cellular component category were the two top abundant subcategories of the two down-regulated patterns (Profile 1 and Profile 0). For the up-regulated pattern of Profile 7, the metabolic process under molecular function was the top subcategories.

All DEGs were subjected to KEGG pathway enrichment analysis, and 36.26 % (5151/14,207) of the DEGs could be annotated. The 20 top KEGG pathways with the highest representation of the DEGs are shown in Table 3. The ribosome (ko03010), plant hormone signal transduction (ko04075), glycolysis/gluconeogenesis (ko00010), starch and sucrose metabolism (ko00500), purine metabolism (ko00230), phenylpropanoid biosynthesis (ko00940), pyrimidine metabolism (ko00240), pyruvate metabolism (ko00620), DNA replication (ko03030) and plantpathogen interaction (ko04626) pathways are significantly enriched. The 372 unigenes among 4366 DEGs (8.52 %) in profile 1, and 122 unigenes accounting for 32.28 % of 378 in profile 0 were annotated to ribosome pathway as the most enriched one, whereas in Profile 7, only 1 unigene accounting for 1.41 % of 71 DEGs, was annotated to this pathway.

**Table 3:** 20 top KEGG pathways with high representation of the DEGs

Pathway	DEGs genes with pathway annotation				Pathway ID
	All profiles (% of 5151)	Profile 1 (% of 4366)	Profile 0 (% of 378)	Profile 7 (% of 71)	
Ribosome	574 (11.14 %)	372 (8.52 %)	122 (32.28 %)	1 (1.41 %)	ko03010
Phagosome	125 (2.43 %)	119 (2.73 %)	3 (0.79 %)	0	ko04145
Citrate cycle (TCA cycle)	80 (1.55 %)	75 (1.72 %)	2 (0.53 %)	3 (4.23 %)	ko00020
Protein processing in endoplasmic reticulum	207 (4.02 %)	190 (4.35 %)	10 (2.65 %)	1 (1.41 %)	ko04141
Proteasome	71 (1.38 %)	71 (1.63 %)	0	0	ko03050
RNA transport	212 (4.12 %)	189 (4.33 %)	10 (2.65 %)	0	ko03013
Oxidative phosphorylation	176 (3.42 %)	153 (3.5 %)	9 (2.38 %)	6 (8.45 %)	ko00190
Glycolysis/Gluconeogenesis	138 (2.68 %)	123 (2.82 %)	9 (2.38 %)	1 (1.41 %)	ko00010
Spliceosome	184 (3.57 %)	180 (4.12 %)	1 (0.26 %)	0	ko03040
Pyruvate metabolism	113 (2.19 %)	97 (2.22 %)	11 (2.91 %)	3 (4.23 %)	ko00620
Carbon fixation in photosynthetic organisms	103 (2 %)	75 (1.72 %)	15 (3.97 %)	3 (4.23 %)	ko00710
Photosynthesis - antenna proteins	46 (0.89 %)	29 (0.66 %)	7 (1.85 %)	0	ko00196
Lysine degradation	22 (0.43 %)	14 (0.32 %)	0	0	ko00310
Glutathione metabolism	78 (1.51 %)	60 (1.37 %)	0	17 (4.5 %)	ko00480
Glyoxylate and dicarboxylate metabolism	67 (1.3 %)	58 (1.33 %)	6 (1.59 %)	0	ko00630
Steroid biosynthesis	24 (0.47 %)	23 (0.53 %)	1 (0.26 %)	0	ko00100
Ascorbate and aldarate metabolism	46 (0.89 %)	37 (0.85 %)	6 (1.59 %)	0	ko00053
Tyrosine metabolism	29 (0.56 %)	23 (0.53 %)	2 (0.53 %)	3 (4.23 %)	ko00350
Nitrogen metabolism	46 (0.89 %)	36 (0.82 %)	5 (1.32 %)	0	ko00910
Valine, leucine and isoleucine degradation	43 (0.83 %)	38 (0.87 %)	1 (0.26 %)	0	ko00280

For the up-regulated pattern of Profile 7, the ten significantly enriched pathways were Phenylpropanoid biosynthesis (ko00940), Metabolic pathways (ko01100), Phenylalanine metabolism (ko00360), Biosynthesis of secondary metabolites (ko01110), Isoquinoline alkaloid biosynthesis (ko00950), Photosynthesis (ko00195), Tyrosine metabolism (ko00350), Plant-pathogen interaction (ko04626), RNA polymerase (ko03020), Oxidative phosphorylation (ko00190) (Table 4). The Metabolic pathways had the largest DEGs number (39), but the Phenylpropanoid biosynthesis has the biggest *P*-value.

**Table 4:** KEGG pathways of Profile 7

Pathway	DEGs genes with pathway annotation (71)	P-value	Pathway ID
Phenylpropanoid biosynthesis	9 (12.68 %)	5.2E-09	ko00940
Metabolic pathways	39 (54.93 %)	1.65E-07	ko01100
Phenylalanine metabolism	6 (8.45 %)	7.08E-06	ko00360
Biosynthesis of secondary metabolites	23 (32.39 %)	8.78E-06	ko01110
Isoquinoline alkaloid biosynthesis	3 (4.23 %)	0.000112	ko00950
Photosynthesis	5 (7.04 %)	0.002424	ko00195
Tyrosine metabolism	3 (4.23 %)	0.003365	ko00350
Plant-pathogen interaction	5 (7.04 %)	0.014188	ko04626
RNA polymerase	3 (4.23 %)	0.016366	ko03020
Oxidative phosphorylation	6 (8.45 %)	0.016567	ko00190

### Analysis of phenylpropanoid biosynthesis pathway genes from *L. minor* unigenes

**Fig. 4:**
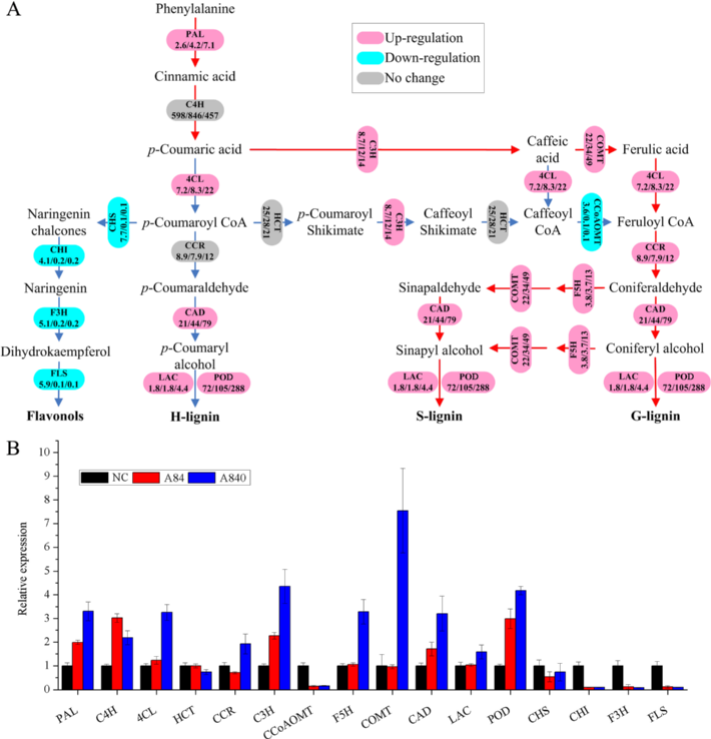
The expression of phenylpropanoid biosynthesis pathway genes from *L. minor* unigenes. **a** lignin and flavonoid biosynthesis in phenylpropanoid biosynthesis pathway (the numbers in the box are the RPKMs of NC, A84 and A840, respectively); **b** qRT-PCR analysis. PAL, Phenylalanine ammonia lyase; C4H, Cinnamic acid 4-hydroxylase; 4CL, 4-hydroxycinnamoyl-CoA ligase; HCT, Hydroxycinnamoyl-CoA: shikimate/quinate hydroxycinnamoyl transferase; CCR, Cinnamoyl-CoA reductase; C3H, p-coumaroyl shikimate 30-hydroxylase; CCoAOMT, Caffeoyl-CoA O-methyltransferase; F5H, Coniferaldehyde/ferulate 5-hydroxylase; COMT, Caffeic acid O-methyltransferase; CAD, Cinnamyl alcohol dehydrogenase; LAC, laccase; POD, Peroxidase; CHS, Chalcone synthase; CHI, Chalcone isomerase; F3H, Flavanone 3-hydroxylas; FLS, Flavonol synthase

In plants, the phenylpropanoid biosynthesis pathway contributes to multiple biosynthetic branches, such as lignin and flavonoid biosynthesis. The expression of transcripts encoding for key enzymes for lignin and flavonoid biosynthesis were analyzed in this study (Fig. 4). The results showed that most of lignin biosynthesis related genes were up-regulated, but not for the expression of transcripts encoding for key enzymes for flavonoid synthesis.

As shown in Fig. 4, in lignin biosynthesis pathway, the genes of PAL (Phenylalanine ammonia-lyase), 4CL (4-hydroxycinnamoyl-CoA ligase), COMT (caffeic acid O-methyltransferase), C3H (p-coumaroyl shikimate 3′-hydroxylase), F5H (coniferaldehyde/ferulate 5-hydroxylase), CCR (cinnamoyl-CoA reductase), LAC (laccase), POD were all up-regulated. HCT (hydroxycinnamoyl-CoA: shikimate/quinate hydroxycinnamoyl transferase) and C4H (cinnamic acid 4-hydroxylase) had almost no change but the gene of CCoAOMT (caffeoyl-CoA O-methyltransferase) showed significant downward trend. This indicated that under NH_4_^+^ stress, shift from caffeoyl CoA to feruloy CoA might be difficult during the biosynthesis of G and S type lignin, which are the main components of monocot lignin [38]. To get feruloy CoA, another way might be potentially utilized which involves the caffeic acid, which can be changed into ferulic acid by COMT, and subsequently changed into feruloy CoA. This mechanism has also been shown to be similar to other monocotyledon like switchgrass [39].

### Expression profiles of oxidative-related and PCD-related genes

**Fig. 5:**
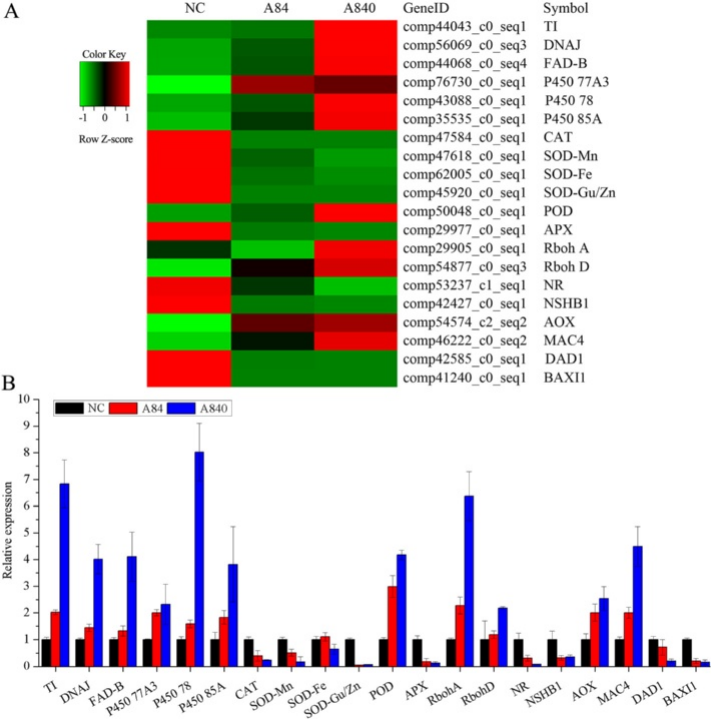
Heatmap (**a**) and qRT-PCR (**b**) analysis of the expression levels of oxidative-related and PCD-related genes. TI, Trypsin/chymotrypsin inhibitor; DNAJ, DNAJ heat shock family protein; FAD-B, FAD-binding domain-containing protein; P450 77A3, Cytochrome P450 77A3; P450 78, Cytochrome P450 85A-like; P450 85A, Cytochrome P450 85A-like; CAT, catalase; SOD-Mn, Superoxide dismutase [Mn]; SOD-Fe, Superoxide dismutase [Fe]; SOD- Cu/Zn, Cu/Zn superoxide dismutase; POD, Peroxidase; APX, L-ascorbate peroxidase; Rboh A, Respiratory burst oxidase homolog protein A; Rboh D, Respiratory burst oxidase protein D; NR, Nitrate reductase; NSHB1, Hemoglobin 1; AOX, Alternative oxidase 1; MAC4, Metacaspase 4; DAD1, Defender against cell death 1; BAXI1, BAX inhibitor 1

Expression of the 14 ROS oxidative-related genes including six oxidative marker genes, six ROS-scavenging genes and two ROS-producing genes are summarized in Fig. 5. The oxidative marker genes included a trypsin/chymotrypsin inhibitor, a DNAJ heat shock protein, a FAD-binding protein and three cytochrome P450 genes, which are regarded as hallmarks for the general oxidative stress response [40, 41]. The ROS-scavenging genes consisted of genes of CAT, SOD and POD.

NADPH oxidases play a key role in generating ROS [42] and have been shown that RbohD and RbohF genes in *Arabidopsis* and RbohA of *Hordeum vulgare* could indeed induce ROS production [43, 44]. In this study, two Rboh-like protein genes, the RbohA and RbohD in *L. minor* were up-regulated under high NH_4_^+^ stress according to the RNA-seq and qRT-PCR results.

On the other hand, three genes indirectly related to oxidative stress were also detected. Under high NH_4_^+^ stress, the nitrate reductase (NR) and non-symbiotic hemoglobin 1 (NSHB1) genes were all down-regulated, but the alternative oxidase 1 (AOX1) gene was up-regulated (Fig. 5).

Metacaspases act as initiators and regulators for programmed cell death (PCD) in plants [45]. In DEGs, a metacaspase gene of MAC4 (metacaspase 4) was significantly up-regulated under NH_4_^+^ stress. Conversely, two inhibitor of PCD, namely BAX inhibitor 1 and DAD1 (defender against cell death 1), were significantly down-regulated.

## Discussion

### NH_4_^+^ toxicity-induced global changes in gene expression in *L. minor*

RNA-seq is a powerful tool that can provide a global overview of gene expression at the transcriptome level. Despite great potentials for both bioenergy applications and environmental studies, only 135 gene sequences of *L. minor* were currently deposited in public databases such as Genbank (accessed 12/10/2015). It is a challenge to do analysis and characterization of *L. minor* RNA-seq dataset without a sequenced genome, and in fact a lack of a sequenced genome in the *Lemna*. Until now, only the chloroplast genome of *L. minor* has been generated [46]. Despite such limitations, careful curation of the sequences and assembly using the robust Trinity Program allowed us to still identify 70,728 unigenes in *L. minor*, and more than 40 % of them were annotated.

The differential expression analysis of RNA-seq data revealed that most of the DEGs were down-regulated under NH_4_^+^ stress. GO and KEGG enrichment analysis revealed that the down-regulated genes (profile 1 and profile 0) mainly categorized into cellular structure and function, metabolic process and gene transcription indicating that some important physiological or “housekeeping” functions might have been inhibited. The same response has been observed in *Arabidopsis* under NH_4_^+^ stress [21]. On the other hand, the up-regulated genes were mainly associated with the metabolic processes, especially the secondary metabolism, such as the Phenylpropanoid biosynthetic pathway (Table 2). It has been suggested that some secondary metabolites play an important role in defenses of abiotic stress [47]. For example, some flavonoids and lignin precursors have been reported to accumulate in response to various abiotic stresses [48].

### NH_4_^+^ toxicity caused oxidative stress and induced cell death

ROS is usually detected in overproduction under abiotic stresses, where it can cause some damages which ultimately results to oxidative stress [49]. Previous studies have shown that *L. minor* could suffer NH_4_^+^ toxicity in high NH_4_^+^ concentrations [16], and that NH_4_^+^ concentration of 56 mg/L could induce oxidative stress [50]. In this study, we further explored the effects of two higher NH_4_^+^ concentrations (84 and 840 mg/L) on oxidative damage of *L. minor*. Like other aquatic plants [51], the accumulated ROS and increased MDA content in *L. minor* in the two NH_4_^+^ treatments indicated that oxidative damage occurring. This is because the increased ROS could induce oxidative stress that contributes to lipid peroxidation and membrane damage, and the MDA has been considered as the indicator of the damage [52]. In addition to the physical evidence, some molecular evidences on NH_4_^+^ toxicity induced damage were also presented produced from the RNA-seq and qRT-PCR analysis (Fig. 5). The expression of some oxidative marker genes like trypsin/chymotrypsin inhibitor, DNAJ heat shock protein and cytochrome P450 genes were enhanced under high NH_4_^+^ stress. Furthermore, the up-regulated ROS-producing genes of RbohA and RbohD also indicated oxidative stress occurring in *L. minor* under NH_4_^+^ toxicity.

The ROS scavenging enzymes play an important role in the plant’s defense system in response to the generation of ROS. Among the enzymatic antioxidants, the enzyme SOD represents the first line of antioxidant defense by transforming O_2_^−^ into H_2_O_2_, and then the APX, POD, and CAT subsequently metabolize H_2_O_2_ [53]. Under NH_4_^+^ toxicity, the SOD activity of *L. minor* increased but not the gene expression of the three types of SOD, indicating a lag from the gene transcription to enzyme action in this 7-day stress treatment. According to Huang’s observations, the activity of SOD decreased until the 14th day under NH_4_^+^ toxicity [50]. The activated SOD could transform O_2_^−^ into H_2_O_2_ but its removal only relies on POD for the gene expression and activities of CAT and APX which all decreased in A840.

Like ROS, nitric oxide (NO) also plays an important role in plant responses to environmental stress, and there are complex networks of interactions between ROS and NO when plants suffer oxidative stress [54]. In plants, NR could reduce nitrate to produce nitrite, as well as reduce nitrite to produce NO, which possesses antioxidant properties and likely to act as a signal in activating ROS-scavenging enzyme activities under oxidative stresses [55]. The non-symbiotic hemoglobin could scavenge NO, thus building a futile cycle with NR [56]. The alternative oxidase can scavenge NO with ROS as the substrates, as well as prevent the production of excess ROS by stabilizing the redox state of the mitochondrial ubiquinone pool [56, 57]. In this study, the NH_4_^+^ is the sole N source in the two stress treatments, the down-regulated *L. minor* NR gene in A84 and A840 indicated the gene might not be activated without nitrate. The NR-mediated NO production might also be suppressed, even though the NSHB1 gene was also down-regulated. The slightly up-regulated AOX1 gene in *L. minor* may be involved in preventing ROS excessive increase under high NH_4_^+^ stress.

ROS is one of the key regulators of PCD that is an active and genetically controlled form of cell death [58]. In this study, except for the ROS, the cell death was also detected in *L. minor* suffering from NH_4_^+^ toxicity by staining. RNA-seq results further showed that a metacaspase gene, MAC4, was significantly up-regulated in the two NH_4_^+^ treatments. In plants, the metacaspase is a discovered gene family that has distant caspase homologs closely related to PCD [59]. The MAC4 of *Arabidopsis* plays a positive regulatory role in abiotic stress-induced PCD [60]. In addition, our results also showed that the PCD inhibiters, like BAX inhibitor 1 [61] and DAD1 [62], significantly decreased in their gene expression. Thus, we can speculate that the NH_4_^+^ toxicity induced PCD of *L. minor*, and that the ROS might play as an intermediate signaling molecule.

### Lignin biosynthesis plays an important role in NH_4_^+^ toxicity resistance

Lignin is the major components of cell wall and the main structure in plant mechanical support and defense system [63]. There are two pathways for lignin biosynthesis in plants, namely of monolignol and phenylpropanoid pathways [64]. And the stimulation of the phenylpropanoid pathway has been considered as a common feature of some abiotic stress response such as drought, salinity, ozone intoxication and heavy metals [63, 65]. Previous studies also showed that both nitrogen deficiency and fertilization (NH_4_NO_3_) could induce a set of genes required for phenylpropanoid metabolism [66, 67]. In this study, the RNA-seq and qRT-PCR results also showed enhanced expression of some key enzyme genes in phenylpropanoid pathway under high NH_4_^+^ stress in *L. minor*. In addition, all up-regulated genes were lignin biosynthesis-related, rather than flavonoid synthesis, which could be due to the antagonistic relationships of the two biosynthetic pathways [68]. However, it could still be suggested that the NH_4_^+^ toxicity could stimulate the phenylpropanoid pathway of *L. minor*, and lead to a shift of metabolism towards lignin.

G and S type lignin are the main components of monocot lignin [39]. In *L. minor*, a series of genes required for the biosynthesis of two types of lignin were up-regulated, including the rate-limiting enzymatic genes in lignin biosynthesis, like *PAL* [69] and *F5H* [70]. The increased lignin synthesis would result to higher lignin content, which together with other antioxidants, could play an important role in limiting ROS production in the apoplast [63]. This mechanism could be one of the reasons why *L. minor* could resist high NH_4_^+^ stress.

## Conclusions

In this study we report the first large transcriptome study carried out in *L. minor* where we have compared physiological and transcriptional responses to NH_4_^+^ toxicity. Evidence from physiological observations, transcriptome and qRT-PCR analysis indicated that NH_4_^+^ toxicity could induce ROS accumulation which in turn results to oxidative damage and later to cell death in *L. minor*. The antioxidant enzyme system was activated under NH_4_^+^ toxicity for ROS scavenging. We also identified that the phenylpropanoid pathway was stimulated under NH_4_^+^ toxicity, and the lignin biosynthesis was also up-regulated in this metabolic pathway. The increased lignin biosynthesis might play an important role in NH_4_^+^ toxicity resistance.

### Ethics approval and consent to participate

Not applicable.

### Consent for publication

Not applicable.

### Availability of data

The dataset is available from the NCBI Sequence Read Archive (SRA). The BioProject and SRA accession are PRJNA302233 and SRP066224, respectively.

## Additional files

Additional file 1: Table S1. Primers used in this paper. (DOCX 17 kb) Additional file 2: Figure S1. Sequencing saturation analysis (A) and distribution of gene coverage (B) in each library. (DOCX 78 kb) Additional file 3: Figure S2. Unigenes matching the 15 top species using BLASTx in the nr database. (DOCX 415 kb) Additional file 4: Figure S3. COG functional classification of all unigenes sequences. 14,172 (20.04 %) unigenes showed significant similarity to sequences in the COG databases and were clustered into 25 categories. (DOCX 124 kb) Additional file 5: Figure S4. Profiles order based on the *P*-value significance of number assigned versus expected. (DOCX 32 kb) Additional file 6: Differentially expressed genes. (XLSX 818 kb)

## Abbreviations

4CL: 4-hydroxycinnamoyl-CoA ligase
AOX: alternative oxidase
APX: ascorbate peroxidase
C3H: p-coumaroyl shikimate 3′-hydroxylase
C4H: cinnamic acid 4-hydroxylase
CAT: catalase
CCoAOMT: caffeoyl-CoA O-methyltransferase
CCR: cinnamoyl-CoA reductase
COG: clusters of orthologous groups of protein
COMT: caffeic acid O-methyltransferase
DAB: 3,3′-diaminobenzidine
DAD1: defender against cell death 1
DEG: differentially expressed gene
F5H: coniferaldehyde/ferulate 5-hydroxylase
GO: gene ontology
HCT: hydroxycinnamoyl-CoA: shikimate/quinate hydroxycinnamoyl transferase
KEGG: Kyoto encyclopedia of genes and genomes
LAC: laccase
MAC4: metacaspase 4
MDA: malondialdehyde
NBT: nitroblue tetrazolium (NBT)
NO: nitric oxide
NR: non-redundant protein database
NR: nitrate reductase
NSHB1: hemoglobin 1
PAL: phenylalanine ammonia-lyase
PCD: programmed cell death
POD: peroxidase
qRT-PCR: quantitative real time PCR
Rboh: respiratory burst oxidase homolog
RNA-seq: RNA sequencing
ROS: reactive oxygen species
RPKM: reads per kb per million reads
SOD: superoxide dismutase
SRA: sequence read archive
STEM: short time-series expression miner software
