# Supplementary material for: Transcriptomic and physiological analysis of common duckweed Lemna minor responses to NH4+ toxicity
Source: BMC Plant Biol. 2016 Apr 18;16:92. doi: 10.1186/s12870-016-0774-8 (PMC4835947; doi:10.1186/s12870-016-0774-8)
Supplement: Additional file 1: Table S1. — Primers used in this paper. (DOCX 17 kb) [file 12870_2016_774_MOESM1_ESM.docx]

**Additional file 1**

**Table S1. Primers used in this paper**

| **Unigene ID** | **Primer sequence** | **Annotation** | **Symbol** |
| --- | --- | --- | --- |
| comp47297_c0_seq1 | Forward: AACACGCTGCTCCAGGGCTA  Reverse: GGGTTTTTCCTTCGGGGGTA | Phenylalanine ammonia-lyase | PAL |
| comp52715_c0_seq1 | Forward: TCCGAACTCCACTCCCT  Reverse: CTCAACCACCGCAATCT | Cinnamate 4-hydroxylase | C4H |
| comp54152_c0_seq1 | Forward: AGGGCTTATCGGCTACA  Reverse: TCCGCTTACATGGGTTC | 4-coumarate-CoA ligase | 4CL |
| comp56955_c0_seq8 | Forward: GTGTTTGCTTAGACGGGATGTT  Reverse: GGGCAGAGTCACCATCACC | hydroxycinnamoyl-Coenzyme A shikimate/quinate hydroxycinnamoyl transferase | HCT |
| comp54512_c0_seq5 | Forward: ATCTCCACGACATCACCTC  Reverse: ACATCAATGCCAGCACAGT | Cinnamoyl-CoA reductase | CCR |
| comp55212_c0_seq1 | Forward: TGGGCAATGGCTGAGGTC  Reverse: TGAGGGGAGTGGGAGGGT | P-coumaroyl-shikimate 3'-hydroxylase | C3H |
| comp351539_c0_seq1 | Forward: AAAAAACACTGGAAATTGGGG  Reverse: TAAAAAGGCATCTGAGGGGAA | caffeoyl-CoA O-methyltransferase | CCoAOMT |
| comp51250_c0_seq2 | Forward: CGAAACGGCAAAACAAGTA  Reverse: GCGGTCGTAGGTGAGGTAA | Ferulate 5-hydroxylase | F5H |
| comp42881_c0_seq4 | Forward: GCTGCTCTGCTCCCCACC  Reverse: GACTCGCCGTCTTCGTTG | Caffeic O-methyltransferase1 | COMT |
| comp53665_c1_seq1 | Forward: CCAGCACGCAGATGAGATTG  Reverse: TGTGGGCGACTACTCCGATG | Aldehyde dehydrogenase family 2 member C4-like | CAD |
| comp43018_c0_seq1 | Forward: TGGCGATTCAAAGTCACCC  Reverse: CGCTTTCCCAGATTTCGG | Laccase-7 | LAC |
| comp50048_c0_seq1 | Forward: AATGCCACGGAAGCCCTAAG  Reverse: CGATTGTATGCCCACCCGAG | Peroxidase | POX |
| comp43995_c0_seq1 | Forward: GAAAAACTCCGAGCCACGA  Reverse: ACCAAACAGGACACCCCAC | Chalcone synthase | CHS |
| comp331281_c0_seq1 | Forward: AGAGAGTGCTGTGAGGGACC  Reverse: TTTGCTGGAAAATGGTAAGTG | Chalcone isomerase | CHI |
| comp18968_c0_seq1 | Forward: AAGAGTTGGAAATGGAGGC  Reverse: ATCGCAAAGCACAAGGTTA | Flavonol synthase/flavanone 3-hydroxylase | F3H |
| comp182973_c0_seq1 | Forward: TAATGGAAAGAAGAACTGGGCT  Reverse: CTGGTCTGTAAGAAGGAGGATTTT | Flavonol synthase/flavanone 3-hydroxylase | FLS |
| comp44043_c0_seq1 | Forward: CTCTCCTCCTCACCACTTCCCTTGC  Reverse: GGTAGCCACCTCAGATTCCTCACGG | Trypsin/chymotrypsin inhibitor | TI |
| comp56069_c0_seq3 | Forward: GACCCGCCGAAAACTCC  Reverse: TCAATCCCCGAAACGCA | DNAJ heat shock family protein | DNAJ |
| comp44068_c0_seq4 | Forward: GCCCTTCCTATCCACAGTCA  Reverse: CAGGCGATGGAGAAGTTTGA | FAD-binding domain-containing protein | FADB |
| comp76730_c0_seq1 | Forward: GAGAGAGAGCGGGAAGAAAA  Reverse: GAAGCCGTGATGAAGGAAGT | Cytochrome P450 77A3 | P450 77A3 |
| comp43088_c0_seq1 | Forward: CGAAGCCACGTCAGGAA  Reverse: GATCGAAGGCGGAAACC | Cytochrome P450, family 78 | P450 78 |
| comp35535_c0_seq1 | Forward: GGGATAGCCCAAAACCA  Reverse: TCGGCGAGACGATAGAA | Cytochrome P450 85A-like | P450 85A |
| comp47584_c0_seq1 | Forward: ATGTTCCCTATCCCACCT  Reverse: ATGAATCGTTCTTGCCTG | Catalase | CAT |
| comp47618_c0_seq1 | Forward: ACACACCCCCAGTCAGCCCT  Reverse: CAGTCCTGGCAGTTGGGCGT | Superoxide dismutase [Mn] | SOD-Mn |
| comp62005_c0_seq1 | Forward: CAGACCCCCATCCTGACCGT  Reverse: CGTTGACGAACTTGTCCAGGTA | Superoxide dismutase [Fe] | SOD-Fe |
| comp45920_c0_seq1 | Forward: TATTGGAAGAGCGGTCGTTGTC  Reverse: TCCACTCAATGTATTATTCCCGC | superoxide dismutase [Gu/Zn] | SOD-Gu/Zn |
| comp50048_c0_seq1 | Forward: AATGCCACGGAAGCCCTAAG  Reverse: CGATTGTATGCCCACCCGAG | peroxidase | POD |
| comp29977_c0_seq1 | Forward: TGCGTAGGAGACCTCTGGGAA  Reverse: CTGGCTCTATCCGCTTCAACC | L-ascorbate peroxidase | APX |
| comp29905_c0_seq1 | Forward: AAACGGGTGCCATTCG  Reverse: CGTGGATGTATCTCGCTGT | Respiratory burst oxidase homolog protein A | Rboh A |
| comp54877_c0_seq3 | Forward: TCGCACTCTTGGTGATTGG  Reverse: AGGGTTAGGGTTGTTGTCG | Respiratory burst oxidase protein D | Rboh D |
| comp46222_c0_seq2 | Forward: AAGGGGAAAATCAGGGTTAGAA  Reverse: ATTTTCAGGGAATCCGCTCTTA | Metacaspase 4 | MAC4 |
| comp42585_c0_seq1 | Forward: CAGAGCAAAAATGGCGAGAAC  Reverse: ACTGCGAAACCCACATACAGA | Defender against cell death 1 | DAD1 |
| comp41240_c0_seq1 | Forward: GCTTTTGGGGCTTATCTTCA  Reverse: ATAAGTAGGGATAGAGAGGAGCCA | BAX inhibitor 1 | BAXI1 |
| Lm18S | Forward: AGAGGAACAGTCGGGGGCATT  Reverse: CGGCATCGTTTACGGTTGAGA |  |  |
