# Supplementary material for: Transcriptomic and physiological analysis of common duckweed Lemna minor responses to NH4+ toxicity
Source: BMC Plant Biol. 2016 Apr 18;16:92. doi: 10.1186/s12870-016-0774-8 (PMC4835947; doi:10.1186/s12870-016-0774-8)
Supplement: Additional file 2: Figure S1. — Sequencing saturation analysis (A) and distribution of gene coverage (B) in each library. (DOCX 78 kb) [file 12870_2016_774_MOESM2_ESM.docx]

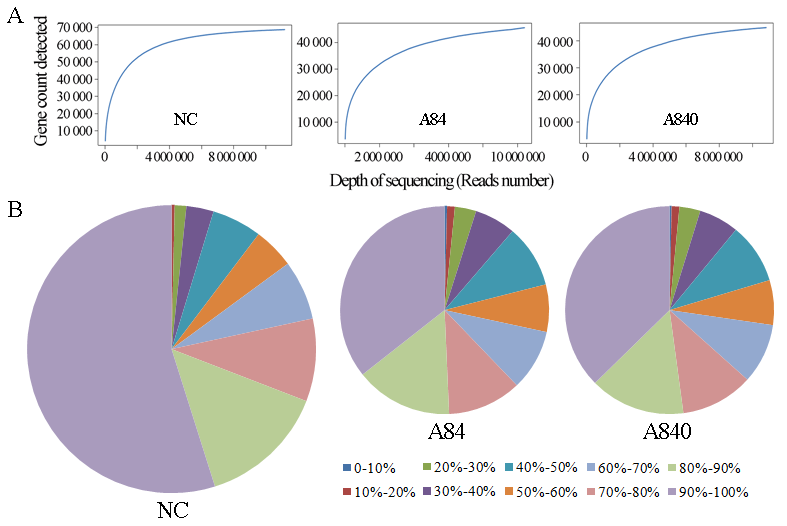


Additional file 2

Figure S1. Sequencing saturation analysis (A) and distribution of gene coverage (B) in each library
