# Supplementary figures and images for: Transcriptomic and physiological analysis of common duckweed Lemna minor responses to NH4+ toxicity
Source: BMC Plant Biol. 2016 Apr 18;16:92. doi: 10.1186/s12870-016-0774-8 (PMC4835947; doi:10.1186/s12870-016-0774-8)

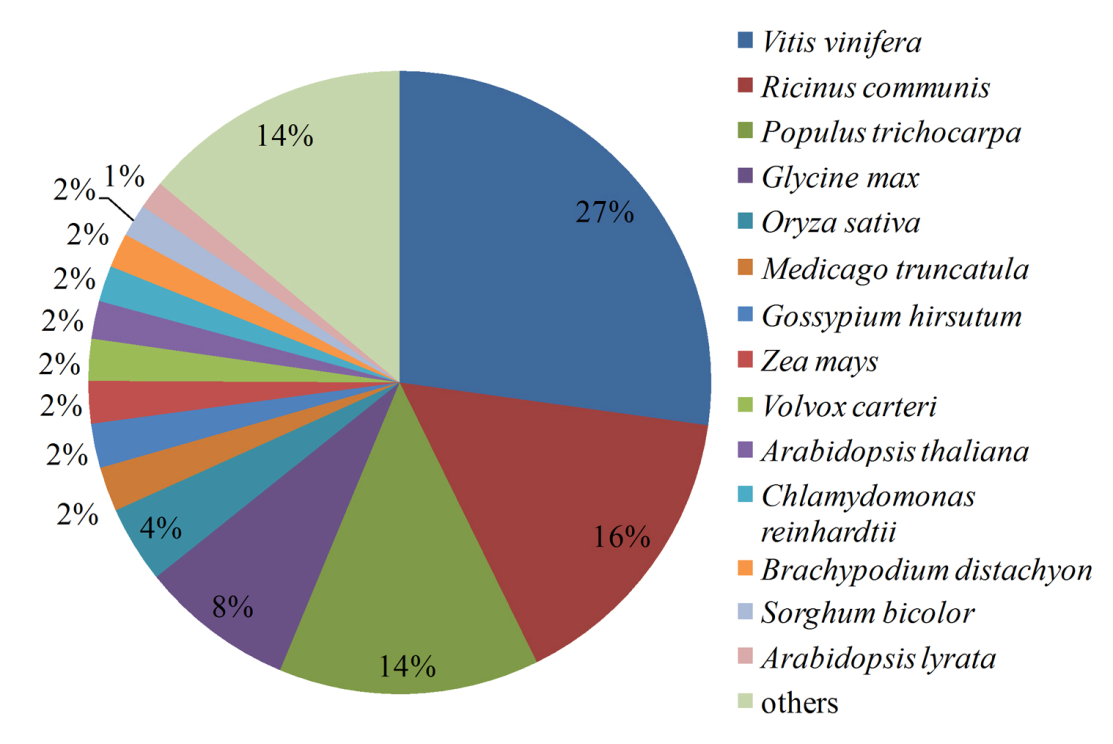


Additional file 3

Figure S2. Unigenes matching the 15 top species using BLASTx in the nr database.

Supplement: Additional file 3: Figure S2. — Unigenes matching the 15 top species using BLASTx in the nr database. (DOCX 415 kb) [file 12870_2016_774_MOESM3_ESM.docx]
