# Supplementary material for: Transcriptomic and physiological analysis of common duckweed Lemna minor responses to NH4+ toxicity
Source: BMC Plant Biol. 2016 Apr 18;16:92. doi: 10.1186/s12870-016-0774-8 (PMC4835947; doi:10.1186/s12870-016-0774-8)
Supplement: Additional file 4: Figure S3. — COG functional classification of all unigenes sequences. 14,172 (20.04 %) unigenes showed significant similarity to sequences in the COG databases and were clustered into 25 categories. (DOCX 124 kb) [file 12870_2016_774_MOESM4_ESM.docx]

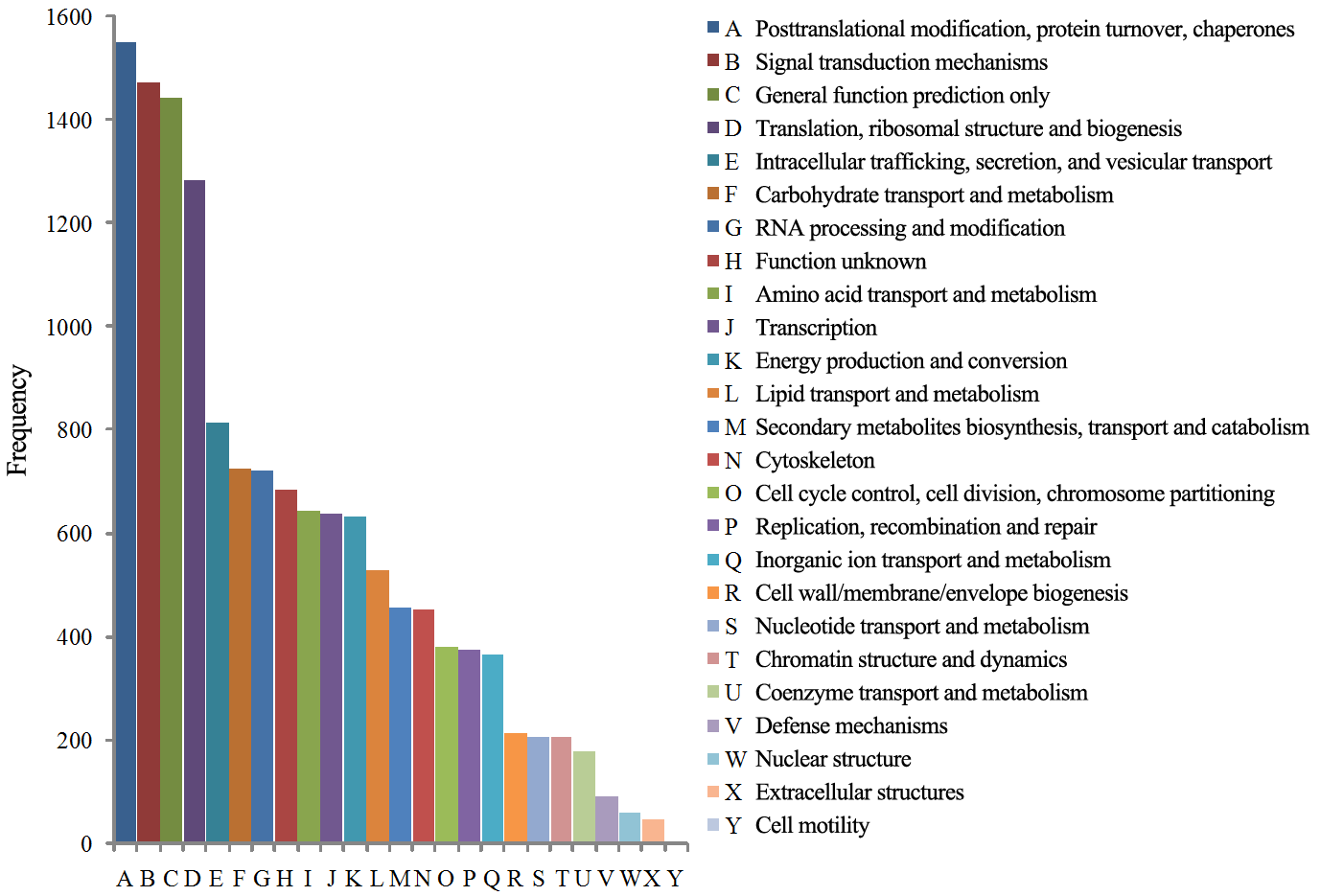


Additional file 4

Figure S3. COG functional classification of all unigenes sequences. 14,172 (20.04 %) unigenes showed significant similarity to sequences in the COG databases and were clustered into 25 categories
