# Supplementary material for: Transcriptomic and physiological analysis of common duckweed Lemna minor responses to NH4+ toxicity
Source: BMC Plant Biol. 2016 Apr 18;16:92. doi: 10.1186/s12870-016-0774-8 (PMC4835947; doi:10.1186/s12870-016-0774-8)
Supplement: Additional file 5: Figure S4. — Profiles order based on the P-value significance of number assigned versus expected. (DOCX 32 kb) [file 12870_2016_774_MOESM5_ESM.docx]

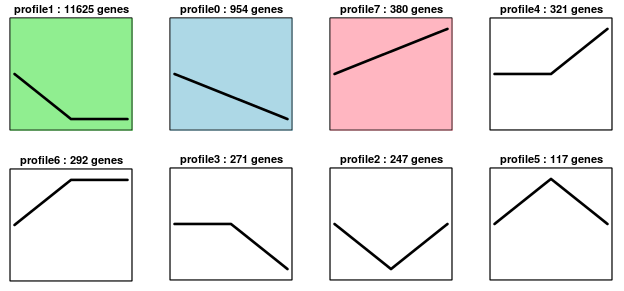


Additional file 5

Figure S4. Profiles order based on the P-value significance of number assigned versus expected.
